# Left Ventricular Free Wall Rupture in Transmural Myocardial Infarction

**DOI:** 10.7759/cureus.1610

**Published:** 2017-08-25

**Authors:** Sidra Khalid, Jyothirmai Seepana, Murtaza Sundhu, Praful Maroo

**Affiliations:** 1 Internal Medicine Residency, Fairview Hospital, Cleveland Clinic, USA; 2 Cardiology, Fairview Hospital, Cleveland Clinic, USA

**Keywords:** left ventricle, free wall rupture, myocardial infarction

## Abstract

Left ventricular free wall rupture (LVFWR) is a grave complication of acute myocardial infarction (MI). We report a case of a 73-year-old male who developed LVFWR five days after a transmural MI. The diagnosis was confirmed with echocardiography, which showed a large pericardial effusion with a clot in the pericardial sac. This case emphasizes that a high index of clinical suspicion for the acute mechanical complications of MI should be present when managing patients with transmural MIs. In addition, stat echocardiography is necessary to diagnose LVFWR and initiate treatment.

## Introduction

Myocardial infarction (MI) is complicated by left ventricular free wall rupture (LVFWR) and pericardial tamponade in 2%-4% of the cases [1]. The free wall rupture site is in the inferolateral (posterior) wall segment (43%), lateral wall (28%), and then the apical wall (24%) followed by other segments at equal frequency [2]. The patients present either with a cardiac arrest, chest pain, and/or arrhythmias. Echocardiography is key for diagnosis. Management involves improving the hemodynamic status of the patient and definitive treatment with surgery. Our patient presented with a transmural MI, in which percutaneous coronary intervention for the lesion did not restore adequate blood flow. The patient had a cardiac arrest and we used echocardiography to diagnose LVFWR. Through our case, we highlight the importance of diagnosing LVFWR in patients with transmural MI.

## Case presentation

**Figure 1:**
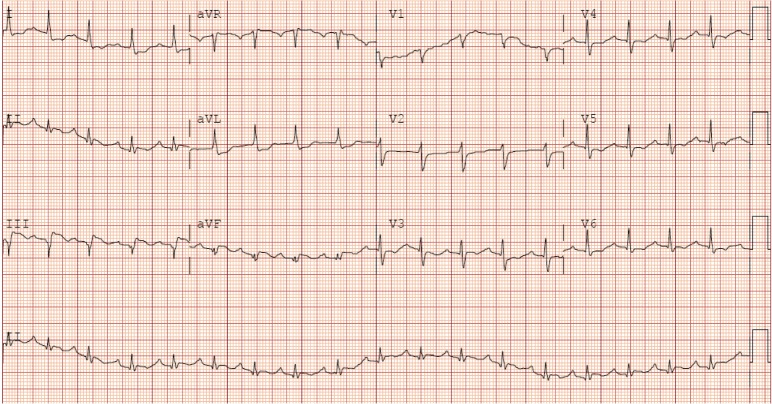
Electrocardiogram - ST elevation in leads III and aVF

**Figure 2:**
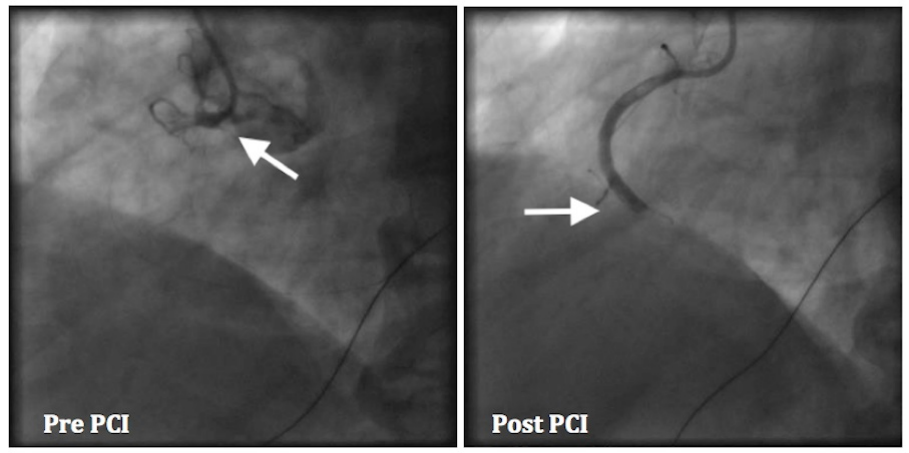
Cardiac catheterization - right coronary artery has total occlusion (arrow). After an intervention with a bare metal stent, the right coronary artery is still occluded (arrow).

A 73-year-old male presented to the emergency department with a sharp, 7/10, nonradiating, midsternal chest pain for four hours. His past medical history included hypertension, hyperlipidemia, and stage III A non-small cell lung carcinoma, in remission for six years after radiation and chemotherapy. On examination, the vitals were stable. The cardiovascular examination was normal and the respiratory exam indicated wheezing. The chest X-ray showed pneumonia in the left lung base. A 12-lead electrocardiogram showed ST segment elevation in leads III and aVF consistent with an acute inferior wall MI (Figure 1). Cardiac catheterization showed a totally occluded proximal right coronary artery (RCA) without a collateral circulation, for which a bare metal stent was placed. Distal to the occlusion in the RCA, there was a large clot burden and aspiration thrombectomy was performed (Figure 2). Intracoronary nitroprusside and tirofiban were infused, followed by a tirofiban drip for one day. The thrombolysis in myocardial infarction (TIMI) flow was 0 and the post-coronary intervention (PCI) TIMI flow was 1. The left ventriculogram showed an ejection fraction of 55% with no wall motion abnormality. The patient was returned to the recovery room in a stable condition. He was started on atorvastatin, metoprolol, and dual antiplatelet therapy. In addition, he was treated for pneumonia with steroids, piperacillin-tazobactam, and azithromycin.

**Figure 3:**
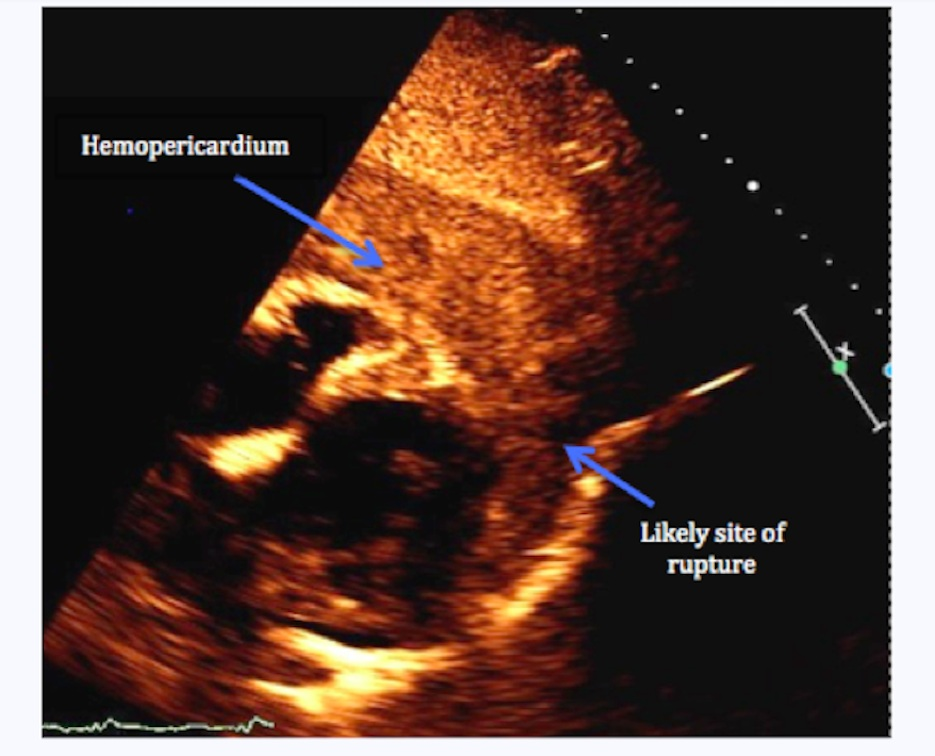
Echocardiogram showing the site of myocardial rupture and the resulting hemopericardium

On Day 5 of his hospital stay, a cardiac arrest occurred, with initial pulseless electrical activity on telemetry. He had a return of spontaneous circulation after two cycles of cardiopulmonary resuscitation (CPR) and was started on norepinephrine for hypotension. The electrocardiogram showed no new ST segment changes. On examination, he was diaphoretic and in no respiratory distress. Jugular venous pressure was mildly elevated and heart sounds were not audible. A stat echocardiogram showed a new large pericardial effusion with a clot in the pericardial sac. There was a muscular dehiscence in the left ventricular apex, indicating a likely site of myocardial rupture (Figure 3). The left ventricle was diffusely hypokinetic with an ejection fraction of 10%-15%. After discussing the poor prognosis with the family, no further intervention was done. The patient passed away five hours after the onset of his symptoms.

## Discussion

An MI complicated by LVFWR carries a high mortality rate. The underlying risk factors causing free wall rupture are: anterior location of the infarct, large transmural infarct, age > 70 years, female sex, no history of previous angina or MI, peak CK-MB above 150 IU/L, ST-segment elevation or Q-wave development on the initial electrocardiogram, and late or failed PCI [3-4].

In a large transmural MI patient without a history of angina or prior MI, there is an increased risk of rupture because there is no collateral circulation present, which would help minimize the development of necrotic tissue [4]. Additionally, the risk of rupture is increased with the use of anti-inflammatory agents post-infarction, such as steroids and nonsteroidal anti-inflammatory drugs. These drugs inhibit fibroblast proliferation and connective tissue regeneration, resulting in impaired wound healing and potential infarct expansion. In contrast, angiotensin-converting enzyme (ACE) inhibitors are protective against myocardial rupture. They reduce the collagenolytic activity of matrix metalloproteinases (MMP), as seen in a study in MMP-9 deficient mice who had decreased the incidence of myocardial rupture [5].

LVFWR is clinically divided into two subtypes, acute and subacute. In the acute form, there is a cardiac arrest due to severe hypotension and electromechanical dissociation secondary to pericardial tamponade. The subacute form presents with persistent or recurrent chest pain, nausea, restlessness, electrocardiogram changes of localized or regional pericarditis, moderate to severe pericardial effusion, sinus bradycardia or nodal arrhythmia, and sudden hypotension or death [3,5].

Echocardiography is crucial for the prompt diagnosis of LVFWR. The finding of pericardial effusion on echocardiography is 100% sensitive, but has low specificity, as the other causes of pericardial effusion after MI are pericarditis, hemorrhage from postinfarcted myocardium, and coronary perforation. Moreover, echocardiography can also show an echogenic layering in the pericardium due to a pericardial thrombus, regional myocardial dilation, and an abnormally thin and akinetic myocardium. In color-flow Doppler echocardiographic evaluation, a color-flow jet visible through a wall defect is diagnostic of LVFWR. However, if a pericardial thrombus covers the rupture site, a color-flow jet would be absent. If the patient is stable, then cardiac computed tomography (CT) and magnetic resonance imaging (MRI) could be considered [4].

Once the diagnosis of myocardial rupture is made, management starts with aggressive volume resuscitation with inotropic and vasopressor support and percutaneous circulatory support, such as intra-aortic balloon counterpulsation and extracorporeal membrane oxygenation. Furthermore, the patient can undergo a surgical repair of the rupture, which includes an infarctectomy or patching. The outcome of surgery depends upon the degree of hemodynamic instability, the location of the rupture, and the weakened state of the surrounding infarcted myocardium. A weakened necrotic surrounding myocardium is a weak anchoring site for sutures in surgical repair. Therefore, when an infarctectomy is performed, it extends to include the viable myocardium, thereby decreasing the size of the left ventricular cavity. In contrast, a Dacron or Teflon patch could be used. These patches are applied with cyanoacrylate glue and cover the area of rupture, the surrounding necrotic tissue, and part of the viable myocardium. Hence, they do not decrease the size of the left ventricular cavity [5]. Additionally, a TachoSil (Takeda, Osaka, Japan) patch could be used in the oozing type of myocardial ruptures because, instead of glue, the patch has fibrinogen and thrombin, which help to apply it [6].

In patients with transmural MI, total coronary occlusion is an essential element in causing a myocardial rupture [5]. In turn, on autopsy, 5%-24% of deaths in MI are due to myocardial rupture, thus making it a high mortality condition [1].

## Conclusions

Our patient had a transmural infarction, but after the revascularization of the lesion, the TIMI flow was low. Therefore, he had the mechanical complication of LVFWR with a cardiac arrest. We were able to diagnose this fatal complication, but no intervention was undertaken, as the prognosis was poor. Therefore, through this case, we would like to suggest that LVFWR is an important differential diagnosis to consider in unstable patients who have had a recent transmural MI in order to have early intervention and improve mortality outcomes.
